# Supplementary material for: Extended Interactions between HIV-1 Viral RNA and tRNALys3 Are Important to Maintain Viral RNA Integrity
Source: Int J Mol Sci. 2020 Dec 23;22(1):58. doi: 10.3390/ijms22010058 (PMC7793103; doi:10.3390/ijms22010058)
Supplement: Supplementary file 1 [file ijms-22-00058-s001.pdf]

Supplementary Material

Extended interactions between HIV-1 viral RNA and $tRNA^{Lys3}$ are important to maintain viral RNA integrity

Thomas Gremminger †, Zhenwei Song †, Juan Ji, Avery Foster, Kexin Weng, and Xiao Heng

Department of Biochemistry, University of Missouri, Columbia, 65211

* Correspondence: hengx@missouri.edu

† These authors contributed equally to this work.

Keywords: HIV-1 PBS, tRNA, reverse transcription, degradation, genome integrity.

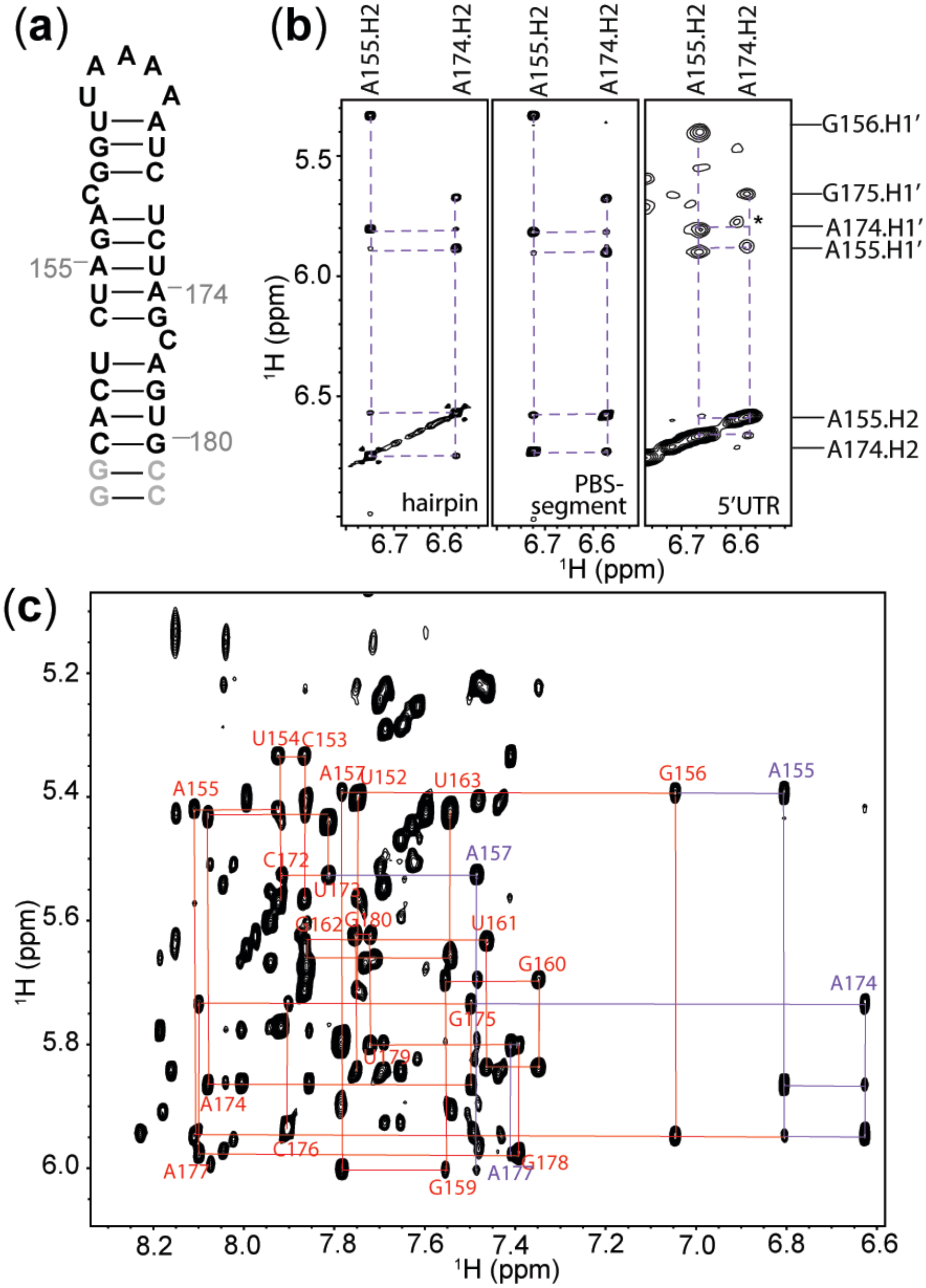

Figure S1: A-rich loop hairpin exhibits similar NOE patterns in all RNA constructs used in this study. (**a**) Secondary structure of A-rich loop hairpin control with non-native bases highlighted in gray. (**b**) Similar NOE patterns were observed in the hairpin control (left), PBS-segment (middle) and the 5’UTR (right). NOESY walk is labeled in purple dashed lines. (**c**) Sequential NOESY walk used for assignment of A-rich loop hairpin control. Red dashed lines indicate walk of H6/H8/H1’ from U152 to A177. Purple dashed lines indicate adenosine H2 resonances .

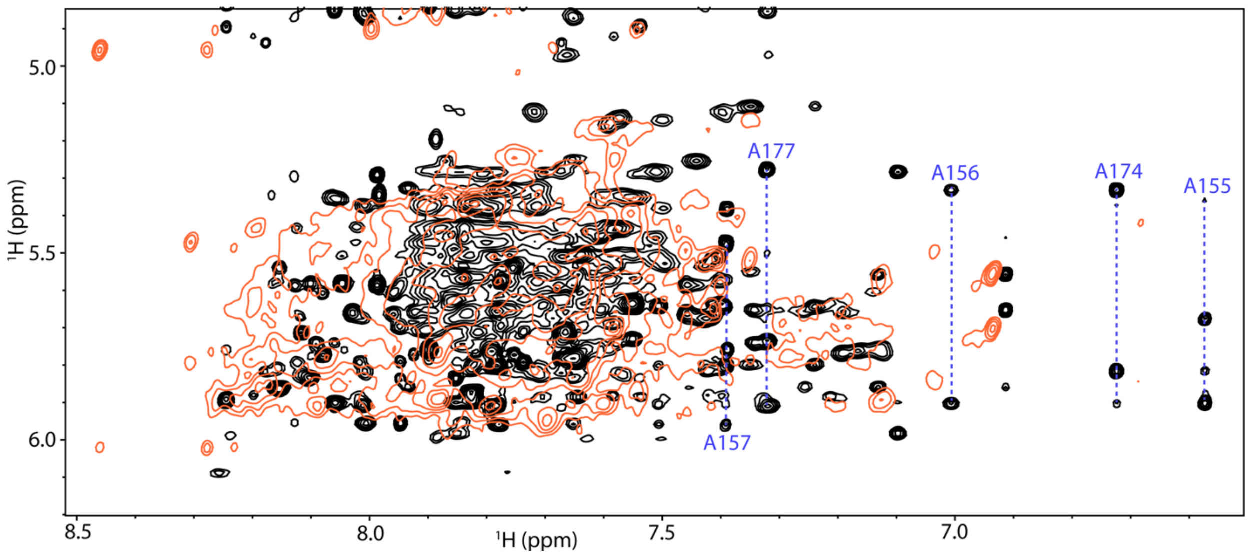

Figure S2. Annealing of tRNA^{Lys3} to PBS-segment disrupted folding of A-rich loop hairpin. Portion of the aromatic region of 2D ^{1}H-^{1}H NOESY spectra with PBS-segment shown in black and PBS-segment: tRNA^{Lys3} complex shown in orange. Resonances of A-rich loop hairpin nucleotides are shown with blue dashed lines.

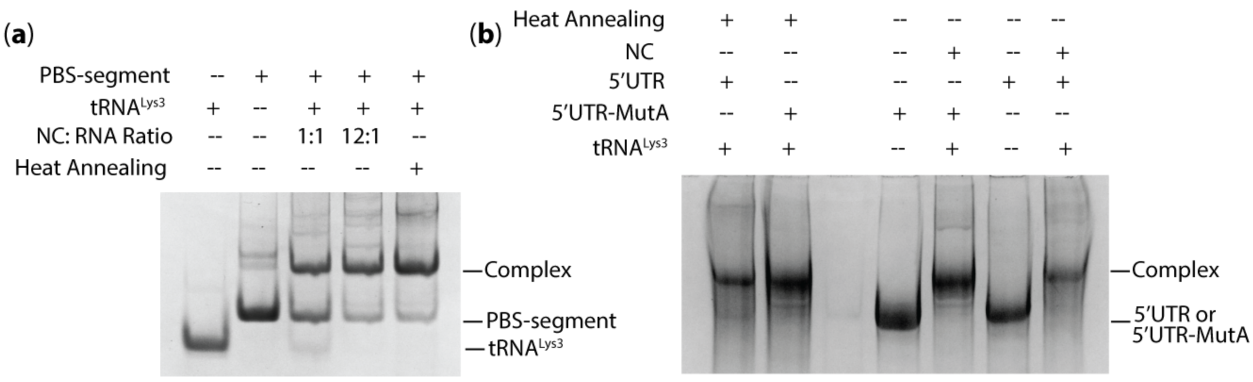

Figure S3. The structure of the MAL vRNA : tRNA^{Lys3} complex is not sensitive to annealing conditions. (**a**) PBS-segment was annealed to tRNA^{Lys3} using NC or heat annealing. Complexes were analyzed using a native polyacrylamide gel. (**b**) The 5'UTR and 5'UTR-MutA were annealed to tRNA^{Lys3} using NC or heat annealing and the complexes were resolved in a native polyacrylamide gel.

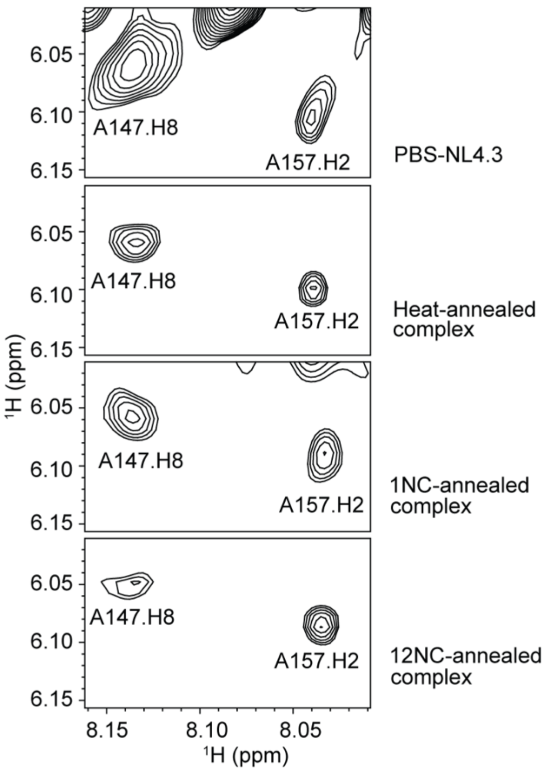

Figure S4. The intensity of A147.H8 decreased upon NC annealing. Portions of 2D ^{1}H-^{1}H NOESY NMR spectra of PBS-NL4.3, heat annealed complex, 1 NC annealed complex (NC: RNA=1:1), and 12 NC annealed complex (NC: RNA=12:1) are shown. The NC proteins were removed by high salt washes prior to NMR data collection. Peak intensities are quantified in table S1.

Table S1: Quantification of peak intensities of 147.H8 and 157.H2 in 2D NOESY spectra of complexes annealed under different conditions.

	147.H8	157.H2	147.H8/157.H2
Heat-annealed complex	1.87	1.78	105%
1NC-annealed complex	1.90	1.77	107%
12NC-annealed complex	1.10	1.88	59%

Table S2. Table of primers used in this study. Notes list which experiment primers were used for.

Primer	Sequence (5’-3’)	Notes
MAL5UTRNL4-3ChimeraUTRF	CTTTTTGCCTGTACTGGTCTCTCTTGTTAGACCAGGTCGAG	Construction of chimeric virus plasmid
MAL5UTRNL4-3ChimeraBackboneR	AGTACAGGCAAAAAGCAGCTGCTTATATGTAGCATCTGAGGG	Construction of chimeric virus plasmid
MAL5UTRNL4-3ChimeraBackboneF	GGTGCGAGAGCGTCGGTATTAAGCGGG	Construction of chimeric virus plasmid
MAL5UTRNL4-3ChimeraUTRR	CGACGCTCTCGCACCCATCTCTCTCCTT	Construction of chimeric virus plasmid
MAL-TAR-F	GCATCTAATACGACTCACTATAGGTCTCTCTTGTTAGAC	Production of template for transcription
MAL-358-R	CGCACCCATCTCTCTCCTTCTAGC	Production of template for transcription
MAL-MutA-F	GTCTTCGGATCTCTAGCAGTGGCGCCCGAACAG	Mutagenesis of MAL plasmid
MAL-MutA-R	TAGAGATCCGAAGACCGTCTAGAGTGGTCTGAGGGATCTCTAGTTACCAG	Mutagenesis of MAL plasmid
3'-ori-R	CTCAAGTCAGAGGTGGCGAAACCCGACAG	Mutagenesis of MAL plasmid
5'-ori-F	CACCTCTGACTTGAGCGTCGATTTTTGTGATGC	Mutagenesis of MAL plasmid
MAL-PBS-F	GCTAGTAATACGACTCACTATAGGCTCTGGTAACTAGAGA	Production of template for transcription
MAL-PBS-R	GGCTCTGGAACTTCCGCTTTCGAGTC	Production of template for transcription
BstZ17I-F	CCTTCACCTGAAATGTGTGTATACAAAATCTAGGCCAGTC	Introducing PBS-M mutant into pNJ4-3 plasmid
BstZ17I-R	CTAGGTATGGTAAATGCAGTATACTTCCTGAAGTCTTTATC	Introducing PBS-M mutant into pNJ4-3 plasmid
NL4-3-PBSM-F	AGACCCTTTTAGTCAGTGTGGTTATCTCTAGCAGTGGCGCCCGAACAG	Introducing PBS-M mutant into pNJ4-3 plasmid
NL4-3-PBSM-R	CACACTGACTAAAAGGGTCTGAGTTATCTCTAGTTACCAGAGTCACAC	Introducing PBS-M mutant into pNJ4-3 plasmid
RT-E478Q-F	CTCAGTTACAAGCAATTCATCTAGCTTTGCAGGATTCG	Introducing E478Q mutant into the pRT-Dual plasmid
RT-E478Q-R	ATGAATTGCTTGTAACTGAGTCTTCTGATTTGTTGTGTC	Introducing E478Q mutant into the pRT-Dual plasmid
pRT-Dual-F	GGCAACGCCAATCAGCAACGACTGTTTGC	Introducing E478Q mutant into the pRT-Dual plasmid
pRT-Dual-R	GCTGATTGGCGTTGCCACCTCCAGTCTG	Introducing E478Q mutant into the pRT-Dual plasmid
hRU5-F2bb	GCCTCAATAAAGCTTGCCTTGA	Quantification of cDNA/ RNA integrity assay
MAL-hRU5-R3	TAGAGTGGTCTGAGGGATCT	Quantification of cDNA/ RNA integrity assay
MAL-hRU5-probe	AGAGTCACACAACAGATGGGCACACACT	Quantification of cDNA/ RNA integrity assay

Gag-F1b	CTAGAACGATTCGCAGTTAATCCT	RNA integrity assay
Gag-R1b	CTATCCTTTGATGCACACAATAGAG	RNA integrity assay
P-HUS-103 (probe)	CATCAGAAGGCTGTAGACAAATACTGG GA	RNA integrity assay
